# Modulatory Effects of Osthole on Lipopolysaccharides-Induced Inflammation in Caco-2 Cell Monolayer and Co-Cultures with THP-1 and THP-1-Derived Macrophages

**DOI:** 10.3390/nu13010123

**Published:** 2020-12-31

**Authors:** Natalia K. Kordulewska, Justyna Topa, Małgorzata Tańska, Anna Cieślińska, Ewa Fiedorowicz, Huub F. J. Savelkoul, Beata Jarmołowska

**Affiliations:** 1 Department of Biochemistry, Faculty of Biology and Biotechnology, University of Warmia and Mazury, 10-719 Olsztyn, Poland; malgorzata.tanska@uwm.edu.pl (M.T.); anna.cieslinska@uwm.edu.pl (A.C.); ewa.kuzbida@uwm.edu.pl (E.F.); bj58@wp.pl (B.J.); 2 Laboratory of Translational Oncology, Intercollegiate Faculty of Biotechnology, Medical University of Gdańsk, 80-211 Gdansk, Poland; 3 Cell Biology and Immunology Group, Wageningen University and Research, 6700 AH Wageningen, The Netherlands; huub.savelkoul@wur.nl

**Keywords:** pro-inflammatory cytokine, interleukin, gene expression, transepithelial electrical resistance, permeability

## Abstract

Lipopolysaccharydes (LPS) are responsible for the intestinal inflammatory reaction, as they may disrupt tight junctions and induce cytokines (CKs) secretion. Osthole has a wide spectrum of pharmacological effects, thus its anti-inflammatory potential in the LPS-treated Caco-2 cell line as well as in Caco-2/THP-1 and Caco-2/macrophages co-cultures was investigated. In brief, Caco-2 cells and co-cultures were incubated with LPS to induce an inflammatory reaction, after which osthole (150–450 ng/mL) was applied to reduce this effect. After 24 h, the level of secreted CKs and changes in gene expression were examined. LPS significantly increased the levels of IL-1β, -6, -8, and TNF-α, while osthole reduced this effect in a concentration-dependent manner, with the most significant decrease when a 450 ng/mL dose was applied (*p* < 0.0001). A similar trend was observed in changes in gene expression, with the significant osthole efficiency at a concentration of 450 ng/μL for IL1R1 and COX-2 (*p* < 0.01) and 300 ng/μL for NF-κB (*p* < 0.001). Osthole increased Caco-2 monolayer permeability, thus if it would ever be considered as a potential drug for minimizing intestinal inflammatory symptoms, its safety should be confirmed in extended in vitro and in vivo studies.

## 1. Introduction

Lipopolysaccharides (LPS) are potent inflammation stimulants that are located on the outer membranes of Gram-negative bacteria. They are responsible for intestinal and systematic inflammatory reactions [1]. Previous studies indicated that LPS disrupt tight junctions (TJ) [1,2,3]. Furthermore, chronic inflammation results in functional impairment of the intestinal barrier to further generate pro-inflammatory cytokines (CKs), which constitutes a vicious cycle. Strategies that alleviate inflammatory response are promising for disease prevention in which maintenance of the intestinal barrier is crucial (such as inflammatory bowel disease (IBD)) [4].

Several in vitro studies discovered natural compounds that can prevent or reverse LPS-induced intestinal barrier damage. Extracts obtained from *Boswellia serrata* and *Curcuma longa* as well as combinations of probiotics and *Chamomilla recutita* extract mitigated LPS-induced epithelial permeability [4,5]. 6-gingerol, a phenolic component in *Zingiber officinale*, also restores intestinal barrier function and suppressed pro-inflammatory responses [6].

Osthole (7-methoxy-8-(3-methylbut-2-en-1-yl)-2H-chromen-2-one), a coumarin bioactive derivative obtained from medicinal plants, is commonly used as an ingredient in herbal medicine and functional foods. Osthole is isolated from the mature fruit of *Cnidium monnieri* and plants of genera *Angelica, Archangelica, Citrus*, and *Clausena.* Fruit of *C. monieri* are commonly applied in traditional Chinese medicine to strengthen the immune system, reduce rheumatic pain, and treat asthma, osteoporosis, and skin diseases [7,8]. Based on in-depth investigations, osthole is revealed to possess a wide range of different pharmacological effects, including anti-allergy [9], anti-inflammatory [10,11], antioxidant [12], hepatoprotective [13,14], neuroprotective [15], anti-osteoporotic [16], and anti-microbial properties [17]. Osthole possess anti-cancer and anti-metastatic activities by inducing cell cycle arrest and apoptosis in many types of cancer, including breast [18,19], ovarian [20], cervival [21], lung [22,23], and gastric cancer [24,25], hepatocellular carcinoma [26], sarcoma [27], glioma [28], and leukemia [29]. Importantly, osthole has been shown to not induce apoptosis and growth inhibition in normal peripheral blood mononuclear cells (PBMCs) and fibroblasts [30]. In our previous research, we confirmed that osthole possesses anti-inflammatory properties in PBMCs isolated from children with diagnosed allergy and autism spectrum disorder (ASD) and from adults with allergy and asthma [9,31,32,33,34,35,36]. However, to date, its anti-inflammatory effect specifically against intestinal inflammation has not been identified.

During the propagation and initiation of IBD and disruption of the intestinal TJ barrier, the mucosal epithelial barrier is compromised, and lymphocyte/macrophages secrete chemokines and pro-inflammatory CKs [37,38,39,40,41,42]. Previously, intestinal inflammation has been studied using animal models [7,38,43,44,45,46]. However, in vitro models are also useful to understand the regulatory mechanisms of anti-inflammatory drugs or food factors [47].

The Caco-2 cell line is widely accepted as a model for human epithelium [40,48,49,50] since Hidalgo et al. reported that they resemble the morphology and function of human intestinal epithelial cells [51]. The Caco-2 model allows the analysis of the biological activity of food components or drugs toward human intestine epithelial cells, reflected in cell proliferation, enzymatic activity, apoptosis, and cytokines secretion. Differentiated Caco-2 monolayer with polarized apical and basolateral TJ is widely used to evaluate the transepithelial transport of chemicals and drugs, including osthole [52,53,54].

In the present study, an in vitro model of intestinal inflammation was established using a co-culture system of human intestinal epithelial Caco-2 cells and monocytic THP-1 cells as well as THP-1-derived macrophages. To evaluate the role of osthole in intestinal inflammation, cells were incubated with LPS alone or in combination as dual mixtures (LPS and osthole). We hypothesize that: (i) tested concentrations of LPS and osthole do not cause a cytotoxic effect on the Caco-2 cell line, (ii) LPS induces changes in secretion of pro-inflammatory CKs (IL-1β, IL-6, IL-8, tumor necrosis factor alpha—TNF-α) as well as affects the expression level of genes encoding interleukin 1 receptor type 1 (IL1R1), nuclear factor kappa B (NF-κB), and cyclooxygenase-2 (COX-2) in the Caco-2 monolayer cell line and co-cultured with THP-1/macrophages, (iii) osthole successively reduces these effects of LPS, (iv) osthole does not affect tight junction integrity in the Caco-2 monolayer and prevents LPS-induced tight junctions disruption, and (v) osthole can be used as a plant-driven substance minimizing the leakage of the intestinal barrier under LPS-induced inflammation.

## 2. Materials and Methods

### 2.1. Caco-2 Cell Culture

The Caco-2 cell line was obtained from American Tissue Culture Collection (ATCC, Manassas, VA, USA) and cultured in T-75 flasks in DMEM (Dulbecco’s modified Eagle’s medium, Sigma-Aldrich, St. Louis, MO, USA, cat. no. D6429) supplemented with 10% fetal bovine serum (Gibco, Thermo Fisher Scientific, Waltham, MA, USA, cat. no. 16000044), 1% nonessential amino acids serum (Gibco, Thermo Fisher Scientific, Waltham, MA, USA, cat. no. 11140050), 0.5% penicillin/streptomycin (Sigma-Aldrich, St. Louis, MO, USA, cat. no. P4333), and 0.1% gentamicin (Sigma-Aldrich, St. Louis, MO, USA, cat. no. G1272). Caco-2 cells were incubated at 37 °C in a 95% humidified atmosphere and 5% CO_2_. The culture medium was changed every 2–3 days, and cells were passaged when confluence reached about 80–90%. The cells used in experiments were between passages 56 and 61.

### 2.2. THP-1 Cell Culture

Human THP-1 monocytic cells (ATCC, Manassas, VA, USA) were cultured in T-75 flasks in RPMI 1640 (Sigma-Aldrich, St. Louis, MO, USA, cat. no R8758) containing 10% FBS, 1% penicillin/streptomycin, and 50µM β-mercaptoethanol (Sigma-Aldrich, St. Louis, MO, USA, cat. no M3148). THP-1 cells were incubated at 37 °C in a 95% humidified atmosphere and 5% CO_2_. Cells were subcultured when they reached a concentration of 8 × 10^5^ cells/mL.

### 2.3. THP-1 Differentiation into Macrophages

THP-1 cells were differentiated into resting macrophages according to the protocol described by Pinto et al. (2020) [55]. Briefly, THP-1 cells were resuspended in a culture medium containing 50 ng/mL phorbol 12-myristate-13-acetate (PMA Sigma-Aldrich, St. Louis, MO, USA, cat. no P8139) for 16 h, followed by 48 h rest in complete RPMI.

### 2.4. Chemicals

LPS from *Escherichia coli* O111:B4 (EC Number 297-473-0, MDL number MFCD00164401) and osthole (CAS Number 484-12-8, MDL number MFCD00076049, PubChem Substance ID 329818938) were obtained from Sigma-Aldrich (St. Louis, MO, USA, cat. no. L4391 and O9265, respectively). Stock solutions were prepared as described in Kordulewska et al. (2015) [34]. LPS were dissolved in water and osthole was dissolved in 96% ethanol (Chempur, Piekary Śląskie Poland, cat. no. 653964200). Solutions were filtered through 0.22 µm pore filters and stored at 4 °C for later dilutions.

### 2.5. Cells Proliferation Analysis

Caco-2 cells proliferation in the presence of different concentrations of tested substances was examined using a cell proliferation ELISA BrdU (colorimetric) kit (Roche Diagnostics, Basel, Switzerland, cat. no. 11647229001). Cells were seeded in culture medium into 96-well plates in a concentration of 5 × 10^3^ cells per well. After 24 h, the medium was removed and replaced with the tested substance solutions and BrdU in the final volume of 100 µL. Cells were incubated for 6, 12, 24, 48, and 72 h. After the incubation, the medium was removed, and cells were dried at 60 °C for 1 h. Plates were coated with parafilm and stored at 4 °C for up to 3 days. Subsequently, the manufacturer’s instructions were followed. Percent of cells were calculated in reference to control (100%, cells seeded in DMEM).

### 2.6. Caco-2 Cells Incubation with Examined Substances

**Figure 1:**
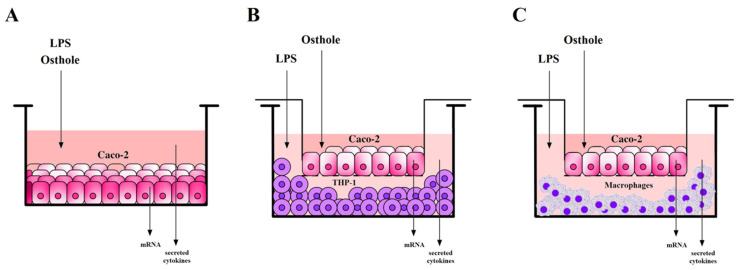
The scheme of experiments performed on Caco-2 cells (**A**) and Caco-2/THP-1 or Caco-2 macrophages co-culture models (**B**,**C**).

Cells were seeded in culture medium into 24 well-plates in a concentration of 2.5 × 10^4^ cells per well. After 24 h, the medium was removed and replaced with fresh DMEM with the addition of LPS (1 µg/mL) or osthole (150–300 ng/mL) in a final volume of 1 mL. After 3 h, osthole in final concentrations of 150 ng/mL, 300 ng/mL, and 450 ng/mL was added to the wells containing LPS. Cells were cultured for 24 h. After that, media were collected, and total RNA was isolated and reverse transcribed. The experiment scheme is shown in Figure 1A.

### 2.7. Caco-2 and THP-1 or Macrophages Co-Culture Models Incubation with Examined Substances

A co-culture model of Caco-2/THP-1 and Caco-2/macrophages was prepared according to the modified protocol described in Kim et al. (2015) [56]. In brief, Caco-2 cells were seeded onto transwell insert plates (Merck St. Louis, MO, USA, cat. no. MCHT06H48) in a concentration of 1.5 × 10^5^ cells/cm^2^ and cultured for 21 days until cells were fully differentiated. The culture medium was changed every 2–3 days. THP-1 or THP-1-derived macrophages were seeded onto 6-well plates (4 × 10^6^ cells/well) and rested for 24 h. After replacing media with complete DMEM, inserts with Caco-2 were added into plates containing THP-1 or macrophages. One microgram per milliliter of LPS was added to the basolateral side, and after 3 h of incubation, different concentrations of osthole (150 ng/mL, 300 ng/mL, and 450 ng/mL) were applied to the apical side of the insert. Osthole was also added to the insert without previous stimulation with LPS. After 24 h of incubation, media from the basolateral side were collected for the CKs secretion analysis, and Caco-2 cells from the insert were collected for total RNA isolation and reverse transcription, as shown in Figure 1B,C.

### 2.8. Post-Culture Media Collection and Isolation of Total RNA

Cell culture plates were centrifuged at 800× *g* for 10 min at 4 °C. Subsequently, post-culture media were collected into tubes and stored at −80 °C for further analysis. Total RNA was isolated according to the protocol described in Kordulewska et al. (2016) [9]. Briefly, cells were lysed in 1 mL TRIzol reagent (Invitrogen, Thermo Fisher Scientific, Waltham, MA, USA, cat. no. 15596026) by repetitive pipetting. Samples were incubated for 5 min at room temperature, then 0.2 mL of chloroform (Chempur, Piekary Śląskie, Poland, cat. no. 112344305) was added. Samples were mixed and centrifuged at 12,000× *g* for 15 min at 4 °C. The aqueous phase was collected and mixed with 0.5 mL of isopropanol to precipitate RNA. Samples were incubated at room temperature for 10 min, then centrifuged at 12,000× *g* for 10 min at 4 °C. The supernatant was discarded, and the RNA pellet was washed with 75% ethanol. Subsequently, the pellet was air-dried and dissolved in diethylpyrocarbonate (DEPC)-treated water. RNA purity was estimated by calculation of the ratio between absorbance at 260 and 280 nm (A_260_/A_280_), with 1.8–2.0 results, and stored at −80 °C for further analysis.

### 2.9. Reverse Transcription

Purified RNA was reverse transcribed by a high-capacity cDNA reverse transcription kit (Applied Biosystems, Thermo Fisher Scientific, Waltham, MA, USA, cat. no. 4368814) according to the manufacturer’s protocol, as described in Kordulewska et al. (2016) [9]. cDNA was stored at −20 °C for further analysis. 

### 2.10. Quantitative Real-Time PCR (qPCR) and Data Analysis

Expression of IL1R1, NF-κB, COX-2, and the human β-actin gene (ACTB) were examined. ACTB was used as a reference gene to normalize disproportion in the mRNA amount. Oligonucleotide primers specific to each gene are listed in Table S1. 

qPCR was performed in the LightCycler 96 real-time PCR system with the FastStart Essential DNA Green Master kit (Roche Diagnostics, Basel, Switzerland, cat. no. 06402712001), as described in Kordulewska et al. (2016) [9]. Five microliters of cDNA was given for reaction and qPCR was performed in triplicate under the following conditions: denaturation at 95 °C for 10 min, amplification and quantification repeated 45 times (95 °C for 20 s, 60/62/63 °C for 20 s, and 72 °C for 20 s with a single fluorescence measurement), melting curve at 60–95 °C with 0.1 °C per second heating rate and continuous fluorescence measurement, and final cooling to 4 °C. A negative control without cDNA and an inter-run calibrator (mix of cDNA of healthy bladder tissue) were included in each assay. Gene expression was analyzed by following Pfaffl (2001) [57]. The results were scaled to the expression level of control, which was determined as one.

### 2.11. Cytokines Level Measurement

Serum IL-1β, -6, -8, and TNF-α levels were examined using enzyme-linked immunosorbent assay (ELISA) kits obtained from Diaclone (Besancon Cedex, France; IL-1β—cat. no. 851.610.001, TNF-α—cat. no. 851.570.001), Mabtech (Nacka Strand, Sweden; IL-6—cat. no. 3460-1H-20), and BD Biosciences (San Jose, CA, USA; IL-8—cat. no. 555244) according to the manufacturers’ protocols. Samples were run in triplicate. Results were standardized by comparison with a standard curve.

### 2.12. Transepithelial Electrical Resistance Measurement

To evaluate tight junction integrity in the Caco-2 monolayer in the presence of tested substances, transepithelial electrical resistance (TEER) measurement was performed using a Millicell ERS-2 volt–ohm meter (Merck St. Louis, MO, USA, cat. no. MERS00002).

Caco-2 cells were seeded onto 0.4 µm pore inserts with an effective growth area of 1.1 cm^2^ (Merck St. Louis, MO, USA, cat. no. MCHT12H48) in concentrations of 1.5 × 10^5^ cells/cm^2^. Cells were cultured for 14 days in complete DMEM, and the medium was changed every second day until cells were fully differentiated and the monolayer reached adequate TEER value (minimum 250 Ω × cm^2^).

TEER measurement was performed immediately after medium replacement with DMEM with LPS, osthole, and its mixtures, and after 1, 2, 6, 24, and 48 h of incubation with tested substances. TEER value was calculated according to the formula described in Srinivasan et al. (2015) [58]. Because TEER values initially differed between wells, measurements at different time points were expressed as a percent of TEER value at time 0 (100%).

### 2.13. Statistical Analysis

Ordinary two-way ANOVA, Tukey’s, and Dunnett’s multiple comparisons tests were used to examine differences between quantitative values. Significance was defined as *p* < 0.05. GraphPad Prism software version 7 (GraphPad Software, San Diego, CA, USA) was used for all statistical analyses.

## 3. Results

### 3.1. Cells Proliferation Analysis

Different concentrations of osthole (150 ng/mL, 300 ng/mL, and 450 ng/mL) and LPS (0.05 µg/mL, 0.1 µg/mL, 0.5 µg/mL, 1 µg/mL, 2 µg/mL) were applied to Caco-2 at 5 time points (6, 12, 24, 48, 72 h). Cell proliferation assays (BrdU) were subsequently performed. It was assumed that maximum inhibition of proliferation can reach 50% of the control, and none of the tested substance variants caused a proliferation decrease to this level (Figure S1). Incubation with tested substances caused a significant increase in proliferation, especially after 6 h of the experiment. Lin et al. (2015) showed that LPS can increase growth via c-Src upregulation [59].

### 3.2. Osthole Reduces the Secretion of Pro-Inflammatory CKs (IL-1β, IL-6, IL-8, and TNF-α) in LPS-Induced Caco-2, Caco-2/THP-1, and Caco-2/Macrophages Co-Culture Model

The secretion of pro-inflammatory CKs into the culture medium was investigated to determine the anti-inflammatory effect of osthole after LPS-induced inflammation in Caco-2 cells and co-culture models. It was determined whether osthole could suppress LPS-induced secretion of IL-1 β, IL-6, IL-8, and TNF-α in Caco-2.

**Figure 2:**
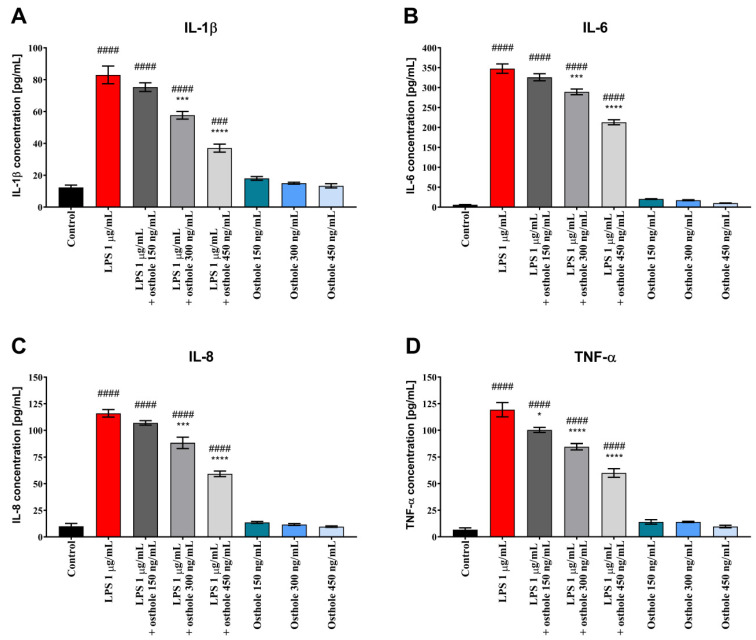
The level of IL-1β (**A**), IL-6 (**B**), IL-8 (**C**) and TNF-α (**D**) after incubation of Caco-2 cells with LPS (1 µg/mL), osthole (150–450 ng/mL) and its mixtures. Horizontal line shows mean and bars depict standard error of mean. Statistically significant differences in comparison to control (###—*p* < 00.01, ####—*p <* 0.0001) and to cells treated with LPS (*—*p* < 0.05, ***—*p* < 0.001, ****—*p <* 0.0001) are marked.

As shown in Figure 2, LPS significantly increased the levels of IL-1 β, IL-6, IL-8, and TNF-α in the medium collected from Caco-2 cultured in 24-well plates (*p* < 0.0001). Osthole reduced this effect in a concentration-dependent manner, with the most significant CKs level reduction when cells were treated with 450 ng/mL of osthole (*p* < 0.001).

**Figure 3:**
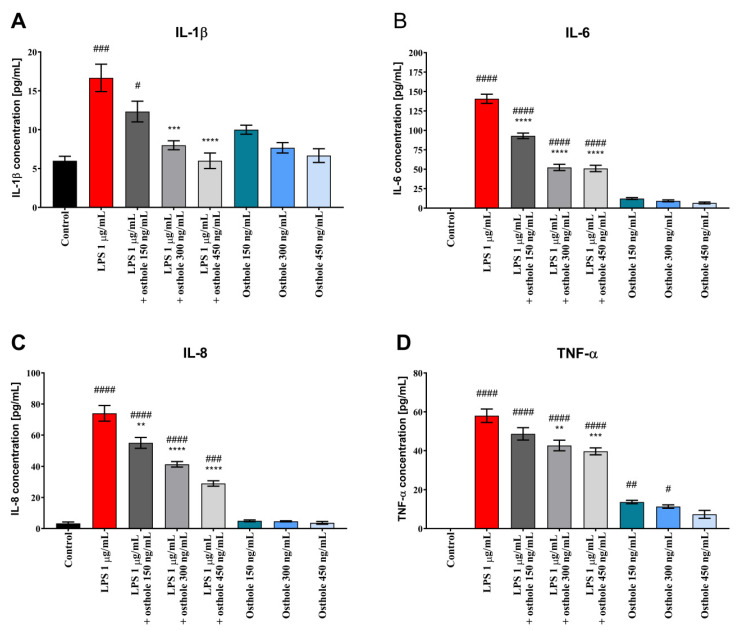
The level of IL-1β (**A**), IL-6 (**B**), IL-8 (**C**) and TNF-α (**D**) in Caco-2/THP-1 co-culture after incubation with LPS (1 µg/mL), osthole (150–450 ng/mL) and its mixtures. Horizontal line shows mean and bars depict standard error of mean. Statistically significant differences in comparison to control (#—*p* < 0.05, ##—*p* < 0.01, ###—*p* < 0.001, ####—*p <* 0.0001) and to cells treated with LPS (**—*p* < 0.01, ***—*p* < 0.001, ****—*p <* 0.0001) are marked.

**Figure 4:**
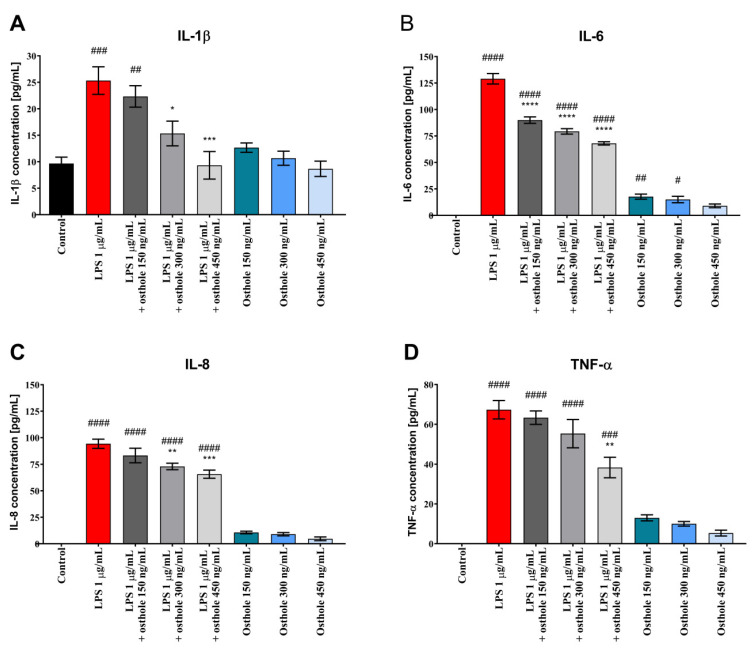
The level of IL-1β (**A**), IL-6 (**B**), IL-8 (**C**) and TNF-α (**D**) in Caco-2/macrophages co-culture after incubation with LPS (1 µg/mL), osthole (150–450 ng/mL) and its mixtures. Horizontal line shows mean and bars depict standard error of mean. Statistically significant differences in comparison to control (#—*p* < 0.05, ##—*p* < 0.01, ###—*p* < 0.001, ####—*p <* 0.0001) and to cells treated with LPS (*—*p* < 0.05, **—*p* < 0.01, ***—*p* < 0.001, ****—*p <* 0.0001) are marked.

Similar trends were observed in Caco-2 cultured with THP-1 and THP-1-derived macrophages. LPS-induced inflammation reflected a significant increase of tested CKs, whereas osthole alleviated this effect, with the most significant decrease when the 450 ng/mL dose was applied (Figure 3 and Figure 4). A significant decrease of IL-6 level was also observed already at the lowest dose of osthole, both in Caco-2/THP-1 and Caco-2/macrophages co-culture models (*p* < 0.0001).

Importantly, osthole alone mostly did not cause an increase in CKs level in Caco-2 monoculture and co-culture models.

### 3.3. Osthole Decreases LPS-Induced Increase of IL-1R1, NF-κB, and COX-2 Expression

**Figure 5:**
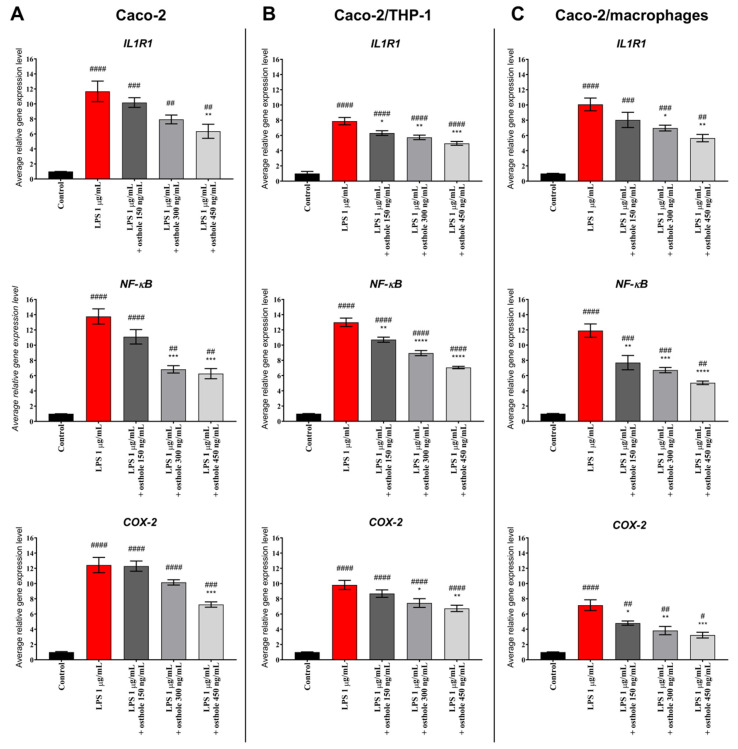
Gene expression level after Caco-2 monoculture (**A**), Caco-2/THP-1 (**B**) and Caco-2/macrohpages (**C**) co-cultures incubation with LPS (1 µg/mL) and with LPS and different concentrations of osthole (150–450 ng/mL). Horizontal line shows mean and bars depict standard error of mean. Gene expression levels were scaled to the control sample (expression level = 1). Statistically significant differences in comparison to control (#—*p* < 0.05, ##—*p* < 0.01, ###—*p* < 0.001, ####—*p <* 0.0001) and to cells treated with LPS (*—*p* < 0.05, **—*p* < 0.01, ***—*p* < 0.001, ****—*p <* 0.0001) are marked.

To assess the anti-inflammatory potential of osthole on mRNA level in Caco-2, changes in gene expression of chosen genes involved in inflammation were analyzed. LPS significantly increased IL1R1, NF-κB, and COX-2 genes expression level in Caco-2 monolayer as well as in Caco-2 cultured with THP-1 and macrophages. In Caco-2, osthole decreased the examined genes’ levels in a concentration-dependent manner, with the significant efficiency at a concentration of 450 ng/μL for IL1R1 and COX-2 (*p* < 0.01) and 300 ng/μL for NF-κB (*p* < 0.001) (Figure 5A). However, Caco-2 co-cultured with THP-1 monocytic cells and macrophages promoted an anti-inflammatory effect, as a significant decrease of the examined genes’ levels was observed in lower osthole concentration (Figure 5B,C).

### 3.4. LPS and Osthole Increase Caco-2 Monolayer Permeability

**Figure 6:**
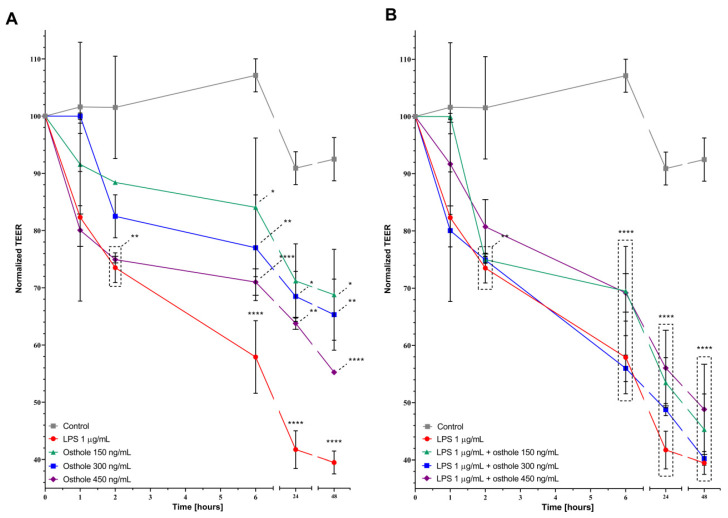
Effects of LPS, osthole (**A**) and its mixtures (**B**) on transepithelial electrical resistance in Caco-2 monolayer). Horizontal line shows mean and bars depict standard error of mean. Statistically significant differences in comparison to control are marked (*—*p* < 0.05, **—*p* < 0.01, ****—*p <* 0.0001).

Tight junction integrity in the Caco-2 monolayer was estimated by TEER measurement. LPS significantly increased monolayer permeability from 6 (*p* < 0.01) to 48 h (*p* < 0.0001) from what was expressed in decreasing TEER value (39.48% after 48 h, Figure 6A). Contrary to the assumed hypothesis, osthole also disturbed monolayer integrity, since after 6 h, TEER value in Caco-2 treated with 450 ng/mL of osthole was 71% of the control (*p* < 0.01). Monolayer permeability was increasing until the end of the experiment and also in cells treated with lower concentrations (150 and 300 ng/mL) of osthole. Even if absolute TEER values in the LPS/osthole-treated Caco-2 monolayer were higher than in Caco-2 cells treated with LPS alone, osthole did not prevent LPS-induced disturbance of tight junctions’ integrity, as there were no significant differences between these measurements (Figure 6B).

## 4. Discussion

Osthole has various beneficial activities, including anti-cancer, anti-inflammation, and antioxidant properties [60]. Even so, there is limited evidence to indicate that osthole can attenuate intestinal inflammation. According to our knowledge, this is the first study that identifies the anti-inflammatory effects of osthole in Caco-2 cells and Caco-2 co-culture models.

In this study, we aimed at exploring the anti-inflammatory properties of osthole in the context of its potential utility as a natural active substance of a drug. For this purpose, we investigated how osthole affects the Caco-2 proliferation, CKs secretion, and gene expression level in LPS-induced Caco-2 as well as in Caco-2/THP-1 and Caco-2/THP-1-derived macrophages.

LPS is a well-known pro-inflammatory factor derived from Gram-negative bacteria. We have shown that LPS, also in the presence of osthole, significantly increased Caco-2 proliferation. Lin et al. (2015) showed that LPS can increase Caco-2 growth via c-Src upregulation in a time-dependent manner [59]. Interestingly, we observed the opposite trend, but our results cannot be directly compared because we applied different LPS concentrations and methods of proliferation assessment.

We demonstrated that after stimulation with LPS, IL-1β, IL-6, IL-8, and TNF-α levels were significantly increased in Caco-2 in comparison to control, suggesting the occurrence of an inflammatory response. After the addition of osthole, secretion of all tested ILs was inhibited. In general, cells treated with osthole alone did not secrete CKs on a significantly higher level. Interestingly, absolute CKs values in co-culture were relatively lower than in Caco-2 monoculture. Kämpfer et al. (2017) previously showed that IL-1β and TNF-α concentration in post-culture media from Caco-2/THP-1 co-culture treated with LPS were significantly lower in comparison to LPS-treated THP-1 monoculture. Absolute CKs values in Caco-2/THP-1 co-culture were also relatively low. Authors also suggested that a down-regulation of the macrophage-driven stress response occurs in the presence of Caco-2 cells [61].

Gene expression analysis showed that osthole inhibits the expression of IL1R1, NF-κB, and COX-2. Obtained results suggest that osthole may suppress the expression of genes involved in inflammation and reduce pro-inflammatory cytokine production in LPS-stimulated cells. Neurath et al. (2014) discussed that the blockade of IL-6 signaling was effective in suppressing chronic inflammation in mouse models, which suggests IL-6 as a potential therapeutic target in IBD. This effect was associated with the induction of T cell apoptosis and the reduced production of pro-inflammatory CKs [62]. We also confirmed that osthole successively reduced IL-6 level in LPS-induced inflammation.

Excessive secretion of TNF-α and IL-1β plays a key role in the pathogenesis of intestinal inflammation, and intestinal epithelial cells can produce soluble mediators that initiate or amplify inflammatory events. Increasing concentration of the IL-1β and TNF-α can cause intestinal mucosa injury and plays one of the most important roles in the occurrence and development of IBD and other inflammatory diseases [62]. Clinical studies have reported that a higher expression of TNF-α was detected in serum and colonic mucosa in ulcerative colitis (UC) patients [63]. TNF-α blockers (e.g., infliximab and adalimumab) have been widely used to treat IBD patients [62]. Our results show that LPS caused a significant increase in TNF-α concentration and that osthole reduced its levels in a concentration-dependent manner. These results suggest that osthole is a potential substance to treat patients with IBD or UC via the suppression of TNF-α.

IL-1β, IL-6, IL-8, and TNF-α were found to stimulate neutrophils recruitment and the secretion of matrix metalloproteinases by intestinal fibroblasts [62]. These findings suggest that these CKs may induce tissue destruction in IBD. Osthole successively decreased secretion of tested CKs. Taken together, the above findings suggest that blockage of IL-1β, IL-6, IL-8, and TNF-α production is of key relevance for IBD therapy.

COX-2 is widely distributed in immune and epithelial cells and directly regulates the secretion of prostaglandin E2 (PGE2). Numerous studies have reported a high level of COX-2 in the local tissues and organs of patients with IBD. High expression of COX-2 on mRNA and protein levels was observed in the colonic mucosa of IBD patients [64]. It has been also reported that LPS upregulates COX-2 expression, which was also confirmed in our research. Our results show that osthole decreased COX-2 expression, suggesting that this substance exhibits anti-inflammatory activity.

IL-1β is implicated in the pathogenesis of intestinal inflammation and stimulates NF-κB activation, which serves as an indicator of cellular inflammation [65], which was also confirmed in our study. We provide evidence that the incubation of LPS-treated cells with osthole can inhibit the secretion of IL-1β in all tested models. Furthermore, we speculate that osthole may decrease IL-1β-induced NF-κB activation. Caco-2 cells upon the stimulation of IL-1 β secretion can synthesize and secret IL-8 via the NF-κB pathway [66].

The pathogenesis of intestinal inflammation is a complex process that involves alterations in gut barrier function and food intolerances, resulting in activation of the innate immune system. However, several lines of evidence suggest that NF-κB activation in mucosal epithelia is a critical event in this process. Anti-inflammatory therapies such as anti-TNF-α antibodies and steroids regulating NF-κB activation are commonly used to treat intestinal inflammation but are also associated with significant side effects. Although elemental diets and specific nutrients have been shown to attenuate gut inflammation, it is unclear whether they act by altering the microbiome, innate immunity, and/or the cellular response to allergy inflammation [67,68]. NF-κB plays a central role in the connection between external pro-inflammatory stimuli and gene expression of inflammatory responses in the nucleus [69], thus interfering with NF- kB activation may particularly be beneficial in diseases related to chronic, low-grade inflammation [70].

**Figure 7:**
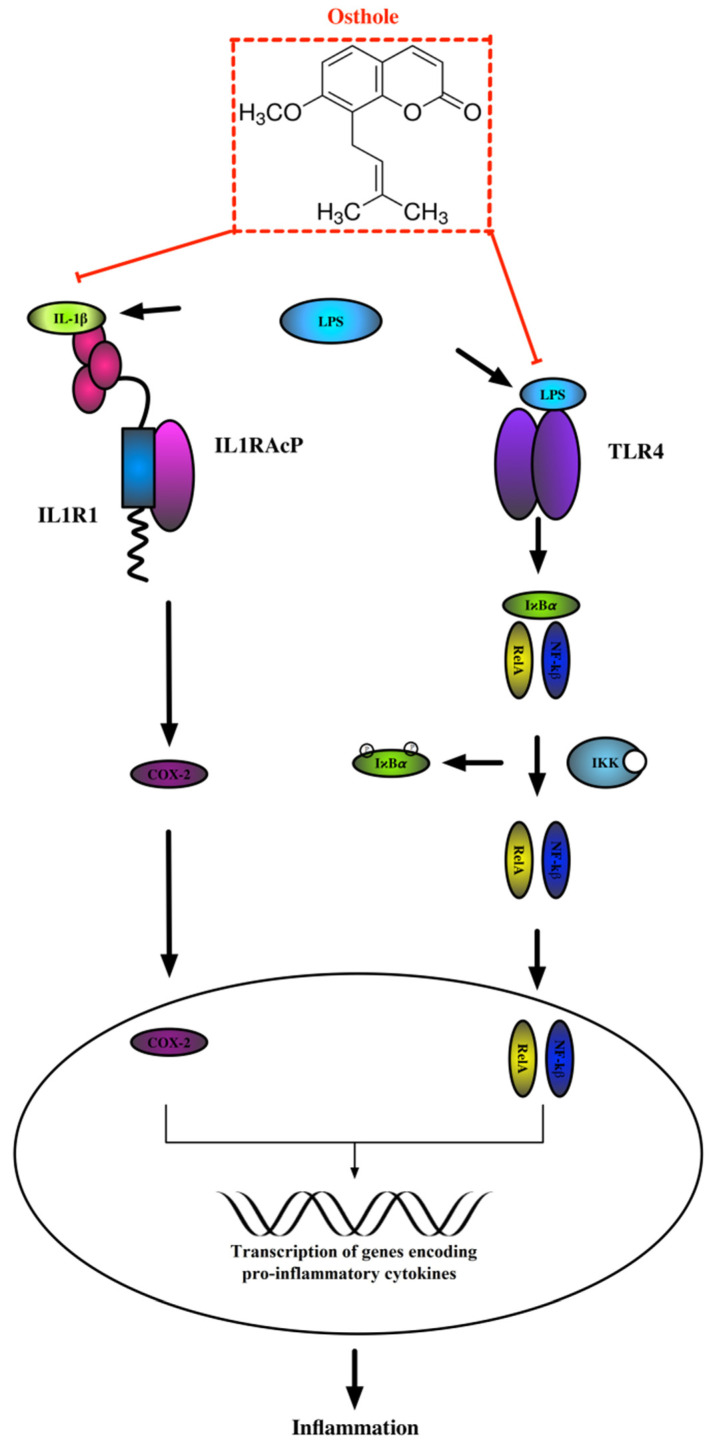
The scheme of LPS-induced inflammatory response.

Pattern recognition and transmembrane receptors play a pivotal role in the initiation of the immune response caused by inflammation inducers (e.g., LPS). One among these, toll-like receptor 4 (TLR4), senses the harmful stimuli and recruits the coordinate activation of transcription factors, including NF-кB [71,72]. NF-кB, as well as IL1R1-activated COX-2, translocates to the nucleus and facilitates transcription of genes encoding pro-inflammatory CKs (Figure 7). We suspect that osthole may act as a regulator in these pathways, and extended studies are needed to uncover the molecular basis of its anti-inflammatory activity.

It has been demonstrated that LPS disrupts tight junctions [73,74]—a finding that was also confirmed in our experiment. Unfortunately, osthole also increased Caco-2 monolayer permeability, and if it would ever be considered as an anti-inflammatory drug, its dosage should be carefully selected.

## 5. Conclusions

Osthole reduced LPS-induced proinflammatory CKs secretion and IL1R1, NF-κB, and COX-2 genes up-regulation and promoted cell migration. While the mechanisms responsible for the observed effects remain to be elucidated, the anti-inflammatory properties of osthole may play an important role. Our data confirmed the potential role of osthole as a protector against intestinal inflammation; thus, if after extended in vivo and in vitro studies its safety would be confirmed, osthole can be considered an anti-inflammatory ingredient in functional foods or nutraceutical formulations.

## Data Availability

The data presented in this study are available on request from the corresponding author. The data are not publicly available due to privacy.

## Supplementary Materials

The following are available online at https://www.mdpi.com/2072-6643/13/1/123/s1, Figure S1: Changes in Caco-2 cell proliferation after incubation with LPS (A), osthole (B), and LPS and osthole (C). The symbols show the mean and bars depict the standard error of the mean. Statistically significant differences in comparison to control (*—*p* < 0.05, **—*p* < 0.01, ***—*p* < 0.001, ****—*p* < 0.0001) are marked. Table S1: Sequences of the oligonucleotide primers specific to examined genes.

## Abbreviations

LPS	Lipopolysaccharides
TJ	Tight junctions
CKs	Cytokines
IBD	Inflammatory bowel disease
PBMCs	Peripheral blood mononuclear cells
ASD	Autism spectrum disorder
TNF-α	Tumor necrosis factor alpha
IL1R1	Interleukin 1 receptor 1
NF-κB	Nuclear factor kappa B
COX-2	Cyclooxygenase-2
ACTB	Actin beta
TEER	Transepithelial electrical resistance
ELISA	enzyme-linked immunosorbent assay
UC	Ulcerative colitis
PGE2	Prostaglandin E2
TLR4	Toll-like receptor 4
