# Supplementary material for: Modulatory Effects of Osthole on Lipopolysaccharides-Induced Inflammation in Caco-2 Cell Monolayer and Co-Cultures with THP-1 and THP-1-Derived Macrophages
Source: Nutrients. 2020 Dec 31;13(1):123. doi: 10.3390/nu13010123 (PMC7824174; doi:10.3390/nu13010123)
Supplement: Supplementary file 1 [file nutrients-13-00123-s001.zip › Supplementary files/Table S1.docx]

**Table S1.** Sequences of the oligonucleotide primers specific to examined genes.

| **Gene** | **GenBank ID** | **Sequence** | **Product**  **lenght (bp)** |
| --- | --- | --- | --- |
| *ACTB* | NM_001101.3 | F: 5’-TCCCTGGAGAAGAGCTACGA-3’ | 194 |
|  |  | R: 5’-AGCACTGTGTTGGCGTACAG-3’ |  |
|  |  | R: 5’-TCAGTCCAGGATGGCTTTG-3’ |  |
| *IL1R1* | NM_000877 | F: 5’-GACAGGGCCTAGCTTTCATTT-3' | 204 |
|  |  | R: 5’-TGGCCAATTTTGTCACTAACC-3’ |  |
| *NF-κB* | M58603.1 | F: 5’-TGGAGTCTGGGAAGGATTTG-3' | 129 |
|  |  | R: 5’-CGAAGCTGGACAAACACAGA-3’ |  |
| *COX-2* | M90100.1 | F: 5’-TGGCTACAAAAGCTGGGAAG-3' | 110 |
|  |  | R: 5’-GCTGCTTTTTACCTTTGACACC-3’ |  |
